# Streptococcus pneumoniae Serotype 23B Causing Asymptomatic Sinusitis Complicated by Endocarditis and Meningitis: Sequela of a Non-vaccine Serotype

**DOI:** 10.7759/cureus.41892

**Published:** 2023-07-14

**Authors:** Saliha Erdem, Dhruvil Patel, Suraj V Patel, Shlok Patel, Shivam Patel, Amrit Kanwar

**Affiliations:** 1 Internal Medicine, Wayne State University School of Medicine, Detroit, USA; 2 Internal Medicine, Ross University School of Medicine, Miramar, USA; 3 Medical School, University of Michigan, Ann Arbor, USA; 4 Medical School, University of South Florida, Tampa, USA; 5 Cardiology, Detroit Medical Center, Detroit, USA

**Keywords:** pneumococcal meningitis, osler's triad, austrian triad, pneumococcal vaccine, pcv13, ppsv23, complicated infective endocarditis, streptococcus pneumoniae meningitis, pneumococcal bacteremia, serotype 23b

## Abstract

We describe a rare case of a *Streptococcus pneumoniae* (*S. pneumoniae*) infection causing mitral valve endocarditis and bacterial meningitis in a previously healthy young adult male in his 20s who presented with altered mentation. Though our patient did not endorse any respiratory issues, we suspected the paranasal sinuses to have been the cryptic primary source of disseminated infection into the respiratory system and meninges due to incidental mucosal thickening being found on imaging. Blood and cerebrospinal fluid analyses and cultures revealed the proliferation of *S. pneumoniae* serotype 23B, despite our patient having previously received appropriate pneumococcal vaccinations in his childhood without delinquency. Ultimately, surgical replacement of the mitral valve, as well as a course of ceftriaxone, was indicated for this patient, in which full resolution of symptoms was achieved upon discharge.

## Introduction

*Streptococcus pneumoniae* (*S. pneumoniae*) is a commensal colonizer of the nasopharynx and commonly causes otitis media, sinusitis, and community-acquired pneumonia with a rate of confirmed infections ranging from 5.16 to 6.11 cases per 100,000 adults in the United States [1]. In patients with *S. pneumoniae*, complications via mucosal invasion, such as infective endocarditis and bacterial meningitis, have been known to occur. However, they are increasingly rare due to widespread vaccination and the current antibiotic prescribing climate [2,3]. Native heart valves, most commonly the aortic valve and less commonly the mitral valve, are well-established sites of endocarditis, which itself has been found to be independently associated with meningitis, carrying a 20.7% mortality rate in a 111-cohort group [4]. With the widespread availability and usage of pneumococcal vaccines, the incidence of invasive pneumococcal infections such as meningitis and endocarditis has decreased; however, there has been a rising trend in the number of non-vaccine pneumococcal serotype infections observed [5,6]. Herein, we present a case of concomitant infective endocarditis and bacterial meningitis in an immunocompetent young adult male with no historical antecedents that would suggest an increased susceptibility toward an acute, disseminated infection of *S. pneumoniae*.

## Case presentation

An adult male in his late 20s with no significant past medical history presented to the emergency department with a fever and a headache. On presentation, the patient appeared responsive and cooperative with initial queries, stating that he recently sought care at an outpatient urgent care facility due to shoulder pain, where he was found to be febrile on the initial assessment. COVID-19 and influenza testing performed on arrival were negative. However, following his urgent care visit, the patient had been feeling feverish and fatigue, along with nasal congestion, thus prompting him to come to the hospital. After the initial interview, the patient began to display waxing-and-waning attentiveness and was soon unable to provide any further case details. His mother was then consulted for the remaining historical details, where she stated that the patient had no recent trauma history, intravenous drug use, or any chronic co-morbidity. Reportedly, the patient had also been experiencing a lack of appetite associated with nausea, vomiting, malaise, and diarrhea, along with mild, non-focal congestion. Additionally, the mother stated that he was not taking any medications; however, she did endorse that he was an active smoker with a history of cannabis consumption and frequent codeine abuse. Urinalysis and urine drug screen performed on admission were unremarkable. Notably, the patient did not appear to have any signs indicative of acute respiratory distress during the initial assessment. His vital signs on admission showed the patient to be hypertensive, tachycardic, and febrile, as shown in Table 1. On physical examination, the patient appeared increasingly drowsy; disoriented to time, place, and situation; and started to have mumbled speech. The patient was able to move all of his extremities without any signs of focal neurological deficits. However, on further examination, he had signs of meningeal irritation. The pain or increased resistance was elicited with passive leg extension (a positive Kernig’s sign), and the patient flexed his knees and hips when his neck was flexed (a positive Brudzinski’s sign). The patient showed no significant findings on a cardiopulmonary physical exam. Given the patient’s medical history and physical examination findings, acute meningitis was strongly suspected, and the patient was admitted for further evaluation and management.

**Table 1:** Vital signs at the time of admission

Vital signs	Results	Reference ranges
Temperature (ºC)	38.3	36.5-37.3
Heart rate (beats per minute)	138	60-100
Respiration rate (breaths per minute)	20	12-20
Blood pressure (mmHg)	156/101	90/60-120/80
SpO_2 _(%)	97 on room air	95-100 on room air

In initial labs, our patient was hyponatremic with a sodium level of 124 mEq/L, which was swiftly resolved following IV fluid administration. However, transaminitis, lactic acidosis, and evident leukocytosis at 13.7 cells/mm3 with a predominance in neutrophils persisted as his only other initial lab abnormalities. Analysis of the cerebrospinal fluid (CSF) showed a pattern extremely suggestive of bacterial meningitis, as shown in Table 2. Testing for HIV and syphilis was also negative. Two sets of blood cultures were obtained and showed gram-positive cocci in chains in both bottles consistent with *S. pneumoniae* serotype 23B (susceptible to ceftriaxone). CSF culture also grew numerous *S. pneumoniae*. Additional testing of CSF was negative for the following: cryptococcal antigen and herpes simplex virus 1&2 polymerase chain reaction (HSV PCR).

**Table 2:** CSF sample analysis results CSF, cerebrospinal fluid; WBC, white blood cell; RBC, red blood cell

CSF laboratory tests	Results	Reference ranges
Opening pressure (cmH_2_O)	29	6-25
Cell count (cells/mm^3^)	100	0-5
WBC CSF (mL)	96	0-5
RBC CSF (mL)	76	0-6
Neutrophils CSF (%)	88	0-6
Lymphocytes CSF (%)	12	40-80
CSF glucose (mg/mL)	<10	50-80
Protein (mg/dL)	396	0.15-0.60
CSF color	Pale yellow	Clear/colorless

**Figure 1:**
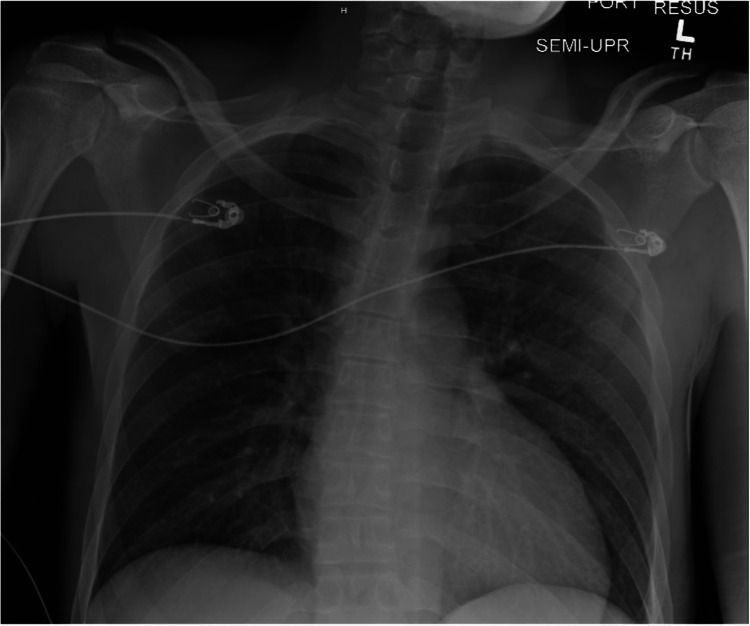
Image of the initial chest X-ray taken on admission There was no evidence of any acute consolidation or localization of bacterial infection that would indicate any pulmonary process from the initial read

**Figure 2:**
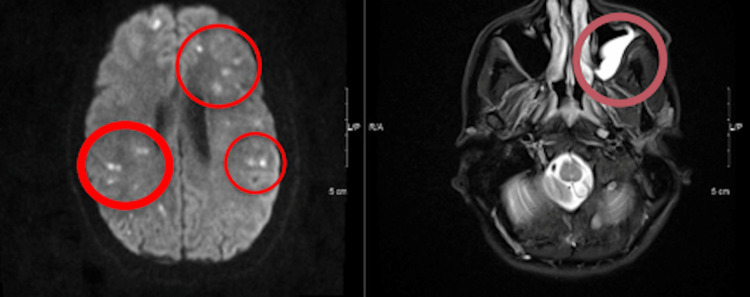
MRI (DWI sequence) demonstrates multiple foci of diffusion restriction (seen within red indicator regions on the left of the image) and (T2-sequence) left maxillary sinus thickening (within a single maroon indicator on the right of the image) DWI, diffusion-weighted imaging

**Figure 3:**
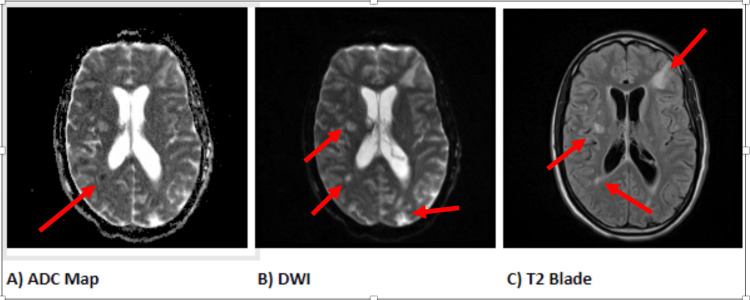
MRI images demonstrating multiple T2 hyperintense foci in deep white matter (A-C), some of which show diffusion restriction with low ADC (A), indicated by arrows ADC, apparent diffusion coefficient

A chest X-ray done early into the admission, as shown in Figure 1, revealed no active acute cardiopulmonary process. A head CT without contrast was done to investigate the signs of nuchal rigidity and meningeal irritation, which revealed mild hydrocephalus with small areas of infarctions seen throughout the cerebral and cerebellar hemispheres with associated edema, as shown in Figures 2, 3. A CTA of the head and neck was subsequently performed. It was negative for mycotic aneurysms and revealed a normal appearance of the major cervical and intracranial arteries, moderate punctate scattering, and patchy hypo-density noted throughout the right cerebral matter bilaterally corresponding to foci of an abnormal T2 hyper-intensity seen on brain MRI, which was read to be consistent with multifocal septic emboli. Furthermore, an MRI of the brain stem showed multiple tiny focal infarctions with embedded foci throughout the cerebral hemispheres that raised suspicion for septic emboli likely of cardiac origin. Incidentally, the MRI brain also showed mucosal thickening in the frontal and anterior ethmoid sinuses as well as in the left ethmoid and maxillary sinuses concerning sinusitis. The evidence of mucosal thickening, along with the lack of infiltrates on the chest X-ray, raised suspicion for an upper respiratory source for pneumococcal bacteremia. An ENT evaluation was requested, where following evaluation, it was reported that there was no evidence of active sinusitis, and thereby, no samples were taken for further study.

**Figure 4:**
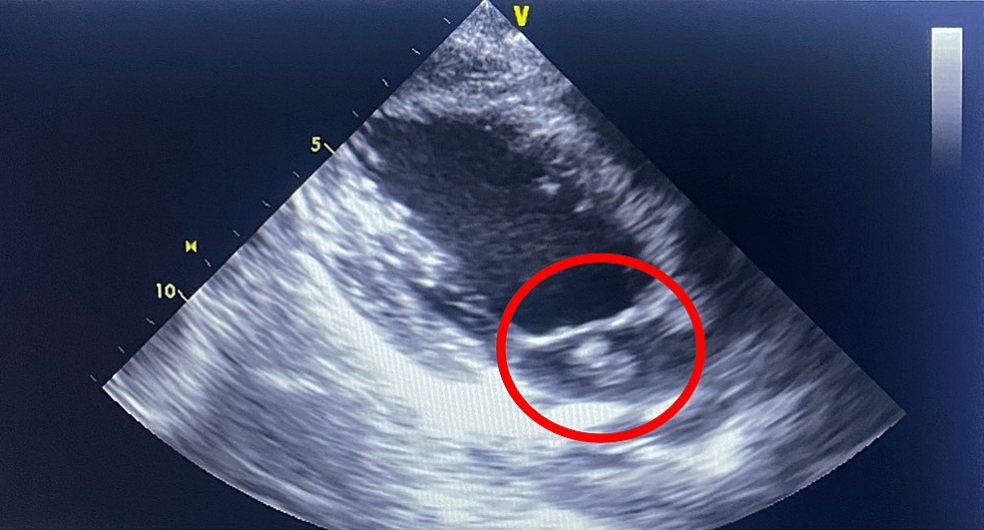
Transthoracic echocardiography indicating large vegetation near the base of the posterior mitral annulus on the left atrial side (indicated in the red circle)

Upon discovery of the potential septic emboli on head and neck radiography, a 2D echocardiogram, as shown in Figure 4, was performed due to concerns for potential cardiac involvement and revealed an EF of 60% with moderate eccentric mitral valve regurgitation. There was a large mobile vegetation that measured 1.1 cm × 1.8 cm near the base of the posterior mitral annulus on the left atrial side and a severely thickened posterior mitral leaflet. The vegetation had a single head present. The recorded pulmonary arterial systolic pressure was 33.3 mmHg. Following this procedure, blood and CSF cultures revealed *S. pneumoniae* serotype 23B (susceptible to ceftriaxone).

The patient was ultimately diagnosed with *S. pneumoniae* endocarditis complicated by bacterial meningitis with cerebritis and ventriculitis. The patient was initially treated empirically with broad-coverage antibiotics, including intravenous vancomycin, ceftriaxone, dexamethasone, and acyclovir, and was later switched solely to ceftriaxone based on CSF testing and the culture results. The dexamethasone was used in a four-day course to prevent inflammatory sequela from bacterial meningitis. The patient was evaluated by cardiothoracic surgery and subsequently underwent a successful mechanical mitral valve replacement. Neurocritical care (NCC) was consulted, and recommendations were obtained regarding the timing of anticoagulation since the patient’s initial MRI brain showed tiny hemorrhages. Therefore, a follow-up MRI brain was obtained a week following the patient’s presentation per NCC recommendations, which did not demonstrate any new foci of bleeding. Therefore, the patient was placed on long-term warfarin anticoagulation with an INR goal of 2.5-3.5 for his new prosthesis following a successful bridging with IV heparin. He remained in the hospital until the target INR levels were achieved, with his mentation slowly returning to baseline levels during that time. No subsequent post-surgical or treatment complications occurred during the remainder of the hospital course, and the patient was discharged after an eight-week antibiotic period was completed. He was discharged in a stable state and has shown continued improvement throughout the follow-up period.

Diagnostic considerations

The initial suspected diagnosis was meningitis, bacterial versus aseptic. Therefore, a spinal tap was performed, and CSF fluid was sent for further analysis. However, alternative diagnoses, in this case, pertained to other causes of altered mental status in a young, previously healthy man, which included drug intoxication, metabolic derangements, stroke, and other causes of sepsis such as urinary tract infection or pulmonary infection. The patient’s chest X-ray and urinalysis were negative, which did not suggest an infection. Urine drug screen and serum toxicology tests ruled out other possible sources of intoxication. The patient did not have any metabolic derangement that could explain altered mental status except for a decreased serum sodium level, which normalized swiftly via IV fluid resuscitation, yet the patient remained mentally altered. Given the CT and MRI head findings that were concerning septic embolization and pan-sinusitis, a 2D echocardiogram was obtained, which showed mitral valve endocarditis. When considered alongside the concomitant bacterial meningitis, it was likely that both the neurological and cardiac findings likely stemmed from a not yet discovered primary source.

## Discussion

In this report, we present a case of pneumococcal bacteremia complicated with both endocarditis and meningitis in a patient with no significant risk factors. Primary endocarditis caused by *S. pneumoniae* is an extremely rare occurrence, with an incidence rate ranging from <0.1% to 3% across various age groups [7-9]. Typically, streptococcal endocarditis manifests with fever, a new heart murmur, and 42% of cases have contaminant pneumonia [4]. In our patient, however, no signs and symptoms consistent with pneumonia were observed. Interestingly, head and neck imaging during the meningitis workup revealed mucosal thickening of the paranasal sinuses, and the patient had symptoms suggestive of sinusitis prior to hospital admission, such as congestion and rhinorrhea. Although para-nasal cultures were not performed at the time, this finding was highly suspicious for the paranasal sinuses being the initial colonization site for the *S. pneumoniae* prior to dissemination. While mucosal thickening was what initially raised clinical suspicion for bacterial proliferation in our case, in which other reports have demonstrated this relationship [10], subsequent studies have shown mucosal thickening in less than 50% of confirmed *S. pneumoniae* infections [11], indicating that the lack of prominent thickening should not exclude sinusitis as a potential diagnosis.

*S. pneumoniae* classically infects native and healthy left-sided heart valves with no previous pathology [4,12,13]. These features were shared in our patient, as he had no history of valvular disease. Additionally, the intra-cardiac vegetations found on the imaging formed primarily at the base of the posterior mitral valve annulus led to subsequent blockage of the brain microvasculature, which led to ischemic infarcts throughout the cerebrum and likely explained our patient’s presenting altered mentation. The results of neural imaging revealed a scattered distribution of focal abnormalities in the cerebellum and cerebral regions, suggesting a pattern that is more likely associated with a secondary introduction process rather than an initial infection and formation. These features urged us to investigate the presence of an intra-cardiac source as the primary means of vegetation formation.

In *S. pneumoniae* infective endocarditis patients, a febrile presentation is very common, with about 39.3-50% of patients having evidence of a newly discovered heart murmur on routine examination [4,14]. Respiratory symptoms may also be evident on presentation as well, with symptoms including shortness of breath and pleuritic chest pain. Our initial examination did not reveal a cardiac murmur or respiratory abnormalities, but it instead showed altered mentation that corroborated findings suspicious for meningitis. While *S. pneumoniae* is the most common cause of bacterial meningitis in younger patients such as ours, nearly 50% of endocarditis cases due to this pathogen in a cohort study also presented with meningitis [4,15]. Moreover, the occurrence of meningitis is limited to approximately 4% of individuals with endocarditis, and *S. pneumoniae* is responsible for about 42.9% of cases where meningitis and endocarditis coexist [16,17]. These data indicate that a presentation of pneumococcal meningitis can also have a primary cardiac source and necessitates evaluation for it, as the initial physical examination findings may be non-specific in determining underlying cardiac involvement.

Also, a vital point to raise is the existence of a triad describing endocarditis, pneumonia, and meningitis due to *S. pneumoniae*, which is referred to as Austrian syndrome/Osler’s triad [18,19]. While uncommon, this devastating condition is associated with rapid mortality and requires immediate consideration if *S. pneumoniae* is a suspected pathogen following patient evaluation [20]. However, our initial chest X-ray evaluation did not find any evidence of preceding pneumonia and thereby ruled out evidence of this condition at the time of admission.

Invasive *S. pneumoniae* infection, while most often observed in children and young adults, has also been reported in older populations. Typically, a history of smoking and alcohol abuse were commonly reported social factors [21] and were present in higher proportions among patients with pneumococcal endocarditis in comparison to other common bacterial causes [10]. Intravenous drug use is a common source of endocarditis, and it should be investigated via a thorough history gathering in young, affected patients. Yet, our patients lacked a suggestive history and had an unremarkable drug screen. Other risk factors for the invasive disease include an immunocompromised state, either from infection or active liver disease often seen with chronic alcohol abuse, which is strongly associated with disseminated pneumococcal infection [22]. We evaluated this potential state early on in our evaluation by performing HIV testing and an STD panel, with neither yielding positive results. Also, the history provided by the mother did not raise suspicion for these features playing a role in our management of the patient.

An important feature to reflect on in our case is the *S. pneumoniae* serotype 23B, which was found to be the causative agent of our patient’s symptoms. The PCV13 vaccine specifically covers 13 serotypes, namely 1, 3, 4, 5, 6A, 6B, 7F, 9V, 14, 18C, 19A, 19F, and 23F [23], and is given from early childhood into adolescence. Therefore, it is possible to see pneumococcal infections that are not covered by vaccination. Stratifying incidence based on the serotype of pneumococcal disease has only been done in one published cohort, which shows serotype 23B to be the greater proportion of new serotypes in the post-PCV13 and PPSV23 vaccine era [5]. Furthermore, the *S. pneumoniae* serotype 23B has been shown to cause more invasive disease than other commonly identified non-vaccine-covered serotypes [24]. This may explain why our patient had two invasive infections concurrently in the absence of any of the common historical risk factors that may predispose to an invasive course of infection.

Central nervous system involvement of an invasive streptococcus infection has also been associated with worsened outcomes and increased mortality. Specifically, the presence of meningitis is found to be the strongest independent risk factor for mortality from *S. pneumoniae* endocarditis [3,4]. Therefore, a manifestation of neurological symptoms in patients with concurrent *S. pneumoniae* endocarditis likely indicates a severe infection that requires conclusive intervention targeted toward the primary source. Surgical valve replacement of the infected valve was by far the most common intervention used in reports of cohorts with similar presentations, but specific indications for when this was done were not reported. Yet, surgery was required much earlier in pneumococcal endocarditis patients than other causes of bacterial endocarditis, attributed to the rapid progression of the bacteria [7,10,25]. Surgery was also associated with decreased mortality when compared to those who did not undergo surgery for pneumococcal endocarditis [7]. These data, while minimal and not in any standard guidelines, do inform the necessity for surgical intervention in those affected with pneumococcal endocarditis and are especially required among patients experiencing neurological sequela.

## Conclusions

The sequela of disseminated *S. pneumoniae* in vaccinated patients presents difficulties in terms of diagnosis and management. Clinicians should be aware that despite vaccination compliance, uncovered serotypes are emerging. Rarely reported in the literature, our case report demonstrates the capacity for a non-vaccine serotype of *S. pneumoniae* to cause infective endocarditis and bacterial meningitis in a fully vaccinated young adult with no historical risk factors. If *S. pneumoniae* and its complications are suspected in a patient who would otherwise not fit the diagnostic profile of an at-risk individual, awareness of infection via a non-vaccine serotype would better serve patient outcomes in terms of accurate diagnosis and appropriate management. In such cases, careful consideration toward identifying a cryptic primary source, as well as consideration for surgical intervention and antibiotic therapy as treatment options, is essential in achieving prompt resolution.
